# Pulmonary Vaccination as a Novel Treatment for Lung Fibrosis

**DOI:** 10.1371/journal.pone.0031299

**Published:** 2012-02-17

**Authors:** Samuel L. Collins, Yee Chan-Li, Robert W. Hallowell, Jonathan D. Powell, Maureen R. Horton

**Affiliations:** 1 Department of Medicine, Johns Hopkins University School of Medicine, Baltimore, Maryland, United States of America; 2 Department of Oncology, Johns Hopkins University School of Medicine, Baltimore, Maryland, United States of America; French National Centre for Scientific Research, France

## Abstract

Pulmonary fibrosis is an untreatable, uniformly fatal disease of unclear etiology that is the result of unremitting chronic inflammation. Recent studies have implicated bone marrow derived fibrocytes and M2 macrophages as playing key roles in propagating fibrosis. While the disease process is characterized by the accumulation of lymphocytes in the lung parenchyma and alveolar space, their role remains unclear. In this report we definitively demonstrate the ability of T cells to regulate lung inflammation leading to fibrosis. Specifically we demonstrate the ability of intranasal vaccinia vaccination to inhibit M2 macrophage generation and fibrocyte recruitment and hence the accumulation of collagen and death due to pulmonary failure. Mechanistically, we demonstrate the ability of lung Th1 cells to prevent fibrosis as vaccinia failed to prevent disease in Rag−/− mice or in mice in which the T cells lacked IFN-γ. Furthermore, vaccination 3 months prior to the initiation of fibrosis was able to mitigate the disease. Our findings clearly demonstrate the role of T cells in regulating pulmonary fibrosis as well as suggest that vaccinia-induced immunotherapy in the lung may prove to be a novel treatment approach to this otherwise fatal disease.

## Introduction

Pulmonary fibrosis is a progressive, fatal disease that primarily affects elderly patients and is due to dysregulated chronic inflammation of the lungs. Currently there are no effective treatments for this disease. A critical barrier to treating this disease is the lack of understanding of the pathophysiology driving the fibrosis. Normally, inflammation in the lungs is a self-limiting process that does not result in scarring or fibrosis. In pulmonary fibrosis however, an unclear initial insult leads to unremitting inflammation characterized by persistent inflammatory cell infiltration, fibroblast recruitment and ensuing fibrosis [1], [2]. While the precise mechanism leading to the development of and persistence of pulmonary fibrosis is unclear, it is clear that this disease is associated with the persistence of pro-inflammatory cytokines and chemokines. Specifically, TNF-α, TGB-β, and both CXC chemokines (IL-8, MIG, IP-10, I-TAC) as well as CC chemokines (MIP-1α, MCP-1) [3]. Additionally, there appears to be a type-2 inflammatory pro-fibrotic response characterized by IL-4 and IL-13 [4], [5], [6], [7].

In both human and animal models, pulmonary fibrosis has been associated with T cell infiltrates in the parenchyma and alveolar space [8], [9]. The increased lymphocytes appear to be predominantly perivascular and in areas of greatest fibrosis and honeycombing [8], [10], [11]. However, the precise role of these cells in either accelerating or inhibiting the development of fibrosis remains unclear. Contradictory data both supports that T cells have the ability to modulate bleomycin induced pulmonary fibrosis as well as that T cells have no role in bleomycin induced lung disease [12], [13], [14], [15], [16], [17]. BALB/c mice, which are naturally resistant to bleomycin-induced lung injury, are rendered susceptible if pre-treated with cyclophosphamide, a drug that depletes T cells [17]. Furthermore, if these cyclophosphamide treated mice are reconstituted with untreated donor BALB/c splenocytes, the resistance to bleomycin is re-established [17]. However, if they are reconstituted with donor spleens depleted of T cells, they remain susceptible [17]. These data support the idea that T cells protect against bleomycin-induced lung injury. Other studies however demonstrate that depletion of all T cells with depleting antibodies can make the disease worse or can have no effect [13], [18]. The data using genetically modified mice are also contradictory in that both SCID and nude mice are equally susceptible to bleomycin as wild type mice [12], [14], [16]. However, CD28 null mice, which cannot fully activate their T cells due to lack of co-stimulation, have a marked attenuation of injury after bleomycin [19].

In as much as T cells are a prominent feature of this disease, these seemingly contradictory data may reflect the fact that different CD4^+^ T cell effector subsets have the ability to either accelerate or mitigate disease. It has become clear that there is great plasticity amongst CD4^+^ T cells with regard to the development of specific effector subsets. Indeed, the pathogenesis of pulmonary fibrosis has been associated with both Th2 and Th17 T cell subsets [7], [20]. Thus we propose a model whereby during normal lung inflammation T cells regulate immunity to prevent fibrosis, while in dysregulated immunity T cell effectors contribute to fibrosis. As such, we posited that the generation of robust Th1 mediated immunity in the lung might serve to inhibit the chronic inflammation leading to fibrosis. In this report we demonstrate that intranasal delivery of live vaccinia vaccine has the ability to arrest and markedly inhibit the development of pulmonary fibrosis. Overall these findings provide insight into the disease process as well as suggest a novel immunotherapeutic approach to treating and preventing Idiopathic Pulmonary Fibrosis.

## Results

### Intranasal Vaccinia enhances survival following bleomycin challenge

**Figure 1:**
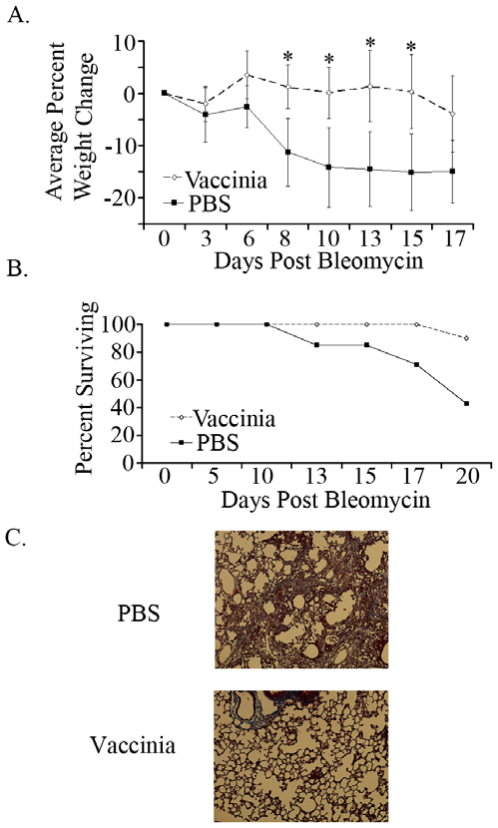
Vaccinia vaccinated mice have enhanced survival following bleomycin administration. A. Weight changes in vaccinia vaccinated and mock (PBS) vaccinated mice following bleomycin administration. B. Survival curves of vaccinia vaccinated and mock (PBS) vaccinated mice following bleomycin administration. C. Masson-Trichrome staining of lungs of PBS and vaccinia treated mice on day 14. Error bars indicate one standard deviation of the mean and (*) indicates statistical significance (p<.05) between groups. All experiments were performed at least three times, at least 10 animals per group per experiment.

In spite of the observation that there is an accumulation of T cells in the lung parenchyma and alveolar space of patients with pulmonary fibrosis, the precise role of T cells in the development of this disease is currently unclear. However, a role for IL-4, IL-13, IL-10 and IL-17 in promoting fibrosis has been described [7]. We hypothesized that the development of a Th1 immune response in the lungs may inhibit the production and activity of these pro-fibrotic cytokines. Vaccinia virus was originally developed as a means of inducing protective immunity against small pox. Vaccination with live vaccinia leads to robust Th1 responses [21]. We utilized a genetically modified vaccinia that contains the full length ovalbumin protein but lacks lytic ability. The ovalbumin was included in the construct as a model cognate antigen and thus allows us to easily track the antigen specific anti-viral immune response. Thus, female C57BL/6 mice were intranasally vaccinated with two million vaccinia particles that express the full length ovalbumin protein or mock vaccinated with PBS. Seven days after the vaccinia vaccination, the mice were given bleomycin by tracheal cut-down and monitored for weight change and survival (Figure 1A & B). As predicted for this model, mice that were initially vaccinated with PBS demonstrated marked weight loss, indicative of disease. After 20 days post-bleomycin, over 50% of the PBS-bleomycin mice had succumbed to pulmonary fibrosis (Figure 1B). However, the mice that were vaccinated with vaccinia were relatively resistant to the disease process (as determined by weight loss) and demonstrated a marked increase in survival (only 10% mortality by Day 20). Histologic examination of the lungs revealed that the decrease in weight and survival in the PBS-bleomycin treated lungs was associated with a marked increase in the development of lung fibrosis (Figure 1C). Thus, intranasal vaccinia vaccination protects mice from the development of bleomycin-induced chronic inflammation leading to the development of fibrosis.

### Intranasal vaccinia vaccination induces a Th1 response in the lungs

We hypothesized that intranasal vaccinia administration would lead to a pulmonary Th1 response that in turn would abrogate the dysregulated inflammation leading to fibrosis. To test this, Ova specific OT-II T cells were transferred into C57BL/6 mice that were then intranasally vaccinated with vaccinia or PBS. Three days later lungs were isolated and single cell suspensions were assessed by FACS. Intranasal vaccinia vaccination led to a marked increase in the Thy1.1^+^ Ova specific T cells to the lungs (Figure S1). Such findings demonstrate the ability of intranasal vaccinia vaccination to generate antigen specific CD4^+^ T cell responses in the lungs.

**Figure 2:**
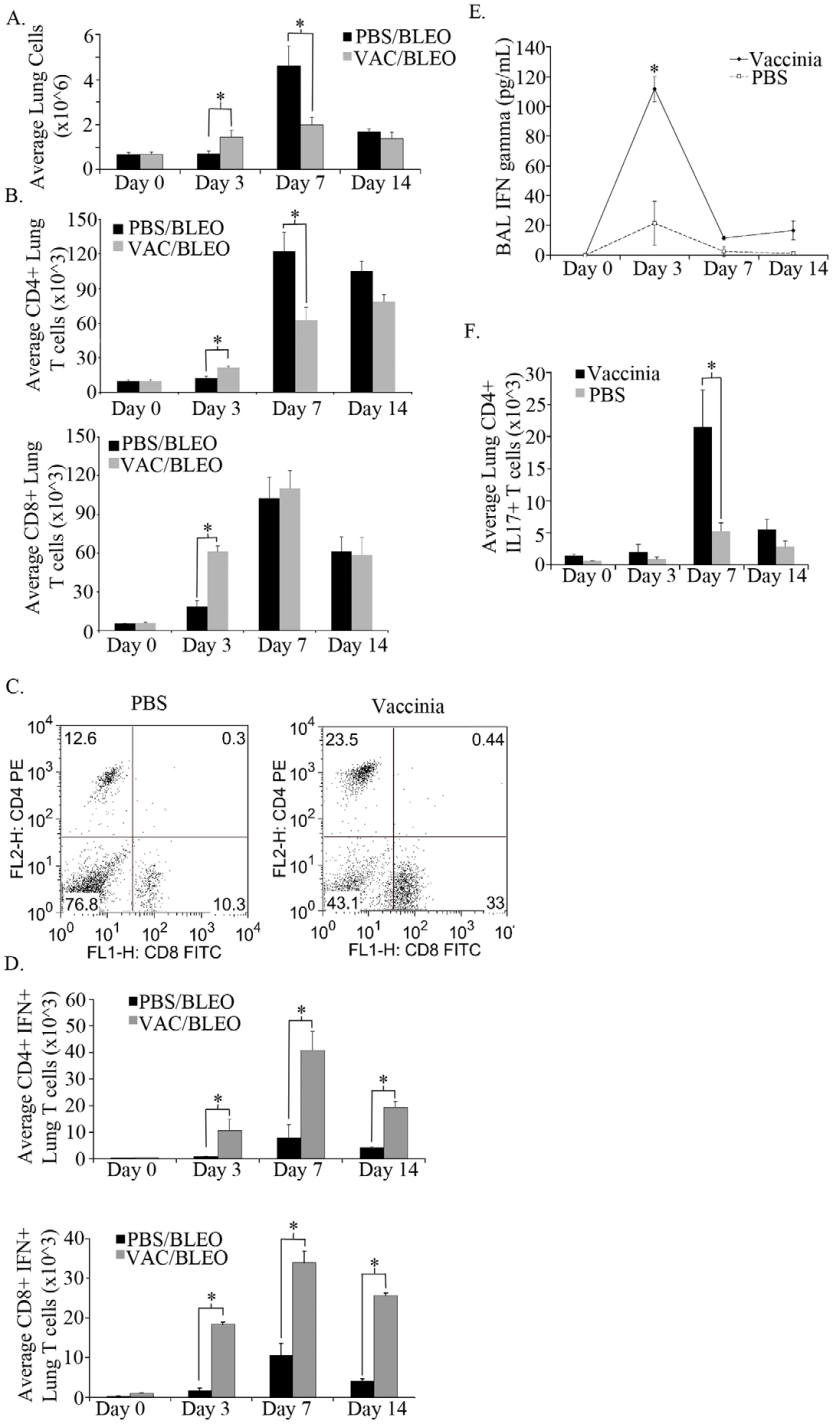
Vaccinia vaccinated mice display greater lung T cell numbers and enhanced function following bleomycin challenge. A. Total lung cell numbers of vaccinia vaccinated and mock (PBS) vaccinated mice following bleomycin administration. B. Total CD4^+^ and CD8^+^ T cells present in the lung following bleomycin administration. C. Flow cytometric analysis of percentages of lung CD4^+^ and CD8^+^ T cells. D. Total lung CD4^+^ IFNγ^+^ and CD8^+^ IFNγ^+^ T cells following bleomycin administration. E. ELISA analysis of IFNγ concentration in bronchial lavage fluid following bleomycin administration. F. Total lung CD4^+^ IL17^+^ T cells following bleomycin administration. Error bars indicate one standard deviation of the mean and (*) indicates statistical significance (p<.05) between groups. All experiments were performed at least three times, at least 10 animals per group per experiment.

Next, we wanted to determine the effector phenotype of these cells when vaccination was followed by bleomycin exposure. Mice were vaccinated on day −7 and challenged with bleomycin on day 0 as previously described, lungs were isolated and single cell suspensions were analyzed for CD4^+^ and CD8^+^ T cells percentages by flow cytometry on days 0, 3, 7, and 14 (Figure 2A). Interestingly, total lung cell numbers were unchanged with or without vaccinia treatment prior to bleomycin administration (day 0). That is, seven days post intranasal vaccination, the total cell numbers from the PBS treated mice and vaccinia treated mice were equivalent. Following bleomycin challenge, vaccinia treated mice had a small but significant increase in total lung cell numbers, however by day seven this trend was reversed and in fact the PBS treated mice displayed increase cellularity in their lungs post-bleomycin exposure. When examining T cells, vaccinia treated mice had greater numbers of CD4^+^ and CD8^+^ T cells on day three following bleomycin than mock (PBS) treated mice (Figure 2B). CD4^+^ T cell numbers remained higher at day seven whereas CD8^+^ T cell numbers were equivalent between vaccinia and mock (PBS) treated mice. Whereas CD8^+^ T cell numbers were equivalent, the percentage of CD8^+^ T cells at day seven was seen to be approximately three times higher in vaccinia treated mice than mock (PBS) vaccinated mice (Figure 2C). Thus, intranasal vaccinia vaccination had a minimal effect on the total number of cells in the lungs of mice post-bleomycin treatment but appeared to slightly alter the kinetics of the appearance of CD4^+^ and CD8^+^ T cells during the acute (0–7 days) inflammatory response to bleomycin.

Since cellularity was not strikingly different between the vaccinated and mock (PBS) vaccinated mice post-bleomycin exposure, we next sought to determine the nature of the inflammatory response in these two groups. We hypothesized that vaccinia would promote a Th1 response in the lungs and therefore we analyzed lung CD4^+^ as well as CD8^+^ T cells for their ability to produce IFNγ. CD4^+^ and CD8^+^ T cells were isolated from the lungs of treated mice and analyzed by FACS for their ability to produce IFN-γ (Figure 2D). T cells derived from the lungs of the vaccinia-treated mice demonstrated robust production of IFN-γ on days 3, 7 and 14 post-bleomycin exposure. In contrast there was very little production of IFN-γ by T cells isolated from the lungs of the unvaccinated mice. Consistent with this increase in IFN-γ producing T cells, there was an increase in IFN-γ in the BAL fluid from the vaccinia vaccinated mice which peaked at day 3 post-bleomycin exposure (Figure 2E). Recent studies have demonstrated a role for IL-17 in promoting the pathogenesis seen in the bleomycin exposure model [20]. As such, we wanted to determine the effect of vaccinia vaccination on the generation of Th17 cells post-bleomycin exposure. Lungs from either mock (PBS) vaccinated or vaccinia treated mice were isolated on days 0, 3, 7, and 14 post bleomycin administration and ICS was performed on CD4^+^ T cells for IL17 (Figure 2F). Consistent with previously published studies, mock (PBS) vaccinated bleomycin exposed mice had increased numbers of IL17^+^ CD4^+^ T cells at all time points analyzed [20]. Alternatively, the induction of Th17 cells was markedly mitigated in the vaccinated mice. Thus, intranasal vaccinia vaccination led to both increased Th1 responses and decreased Th17 responses in the lungs post bleomycin-exposure.

### Vaccinia vaccination promotes an M1 macrophage response

Macrophages are present in significant numbers in the normal lung and increase dramatically upon lung inflammation. Indeed, macrophages are believed to play a critical role in promoting the chronic inflammation leading to lung fibrosis. Specifically, recent studies have suggested that M2 macrophages play a role in promoting the development of fibrosis [22], [23]. The development of M2 macrophages is associated with a Th2 environment including the profibrotic chemokines IL-4 and IL-13 [24]. Thus we wanted to determine the effect of vaccinia vaccination on M2 macrophage development.

**Figure 3:**
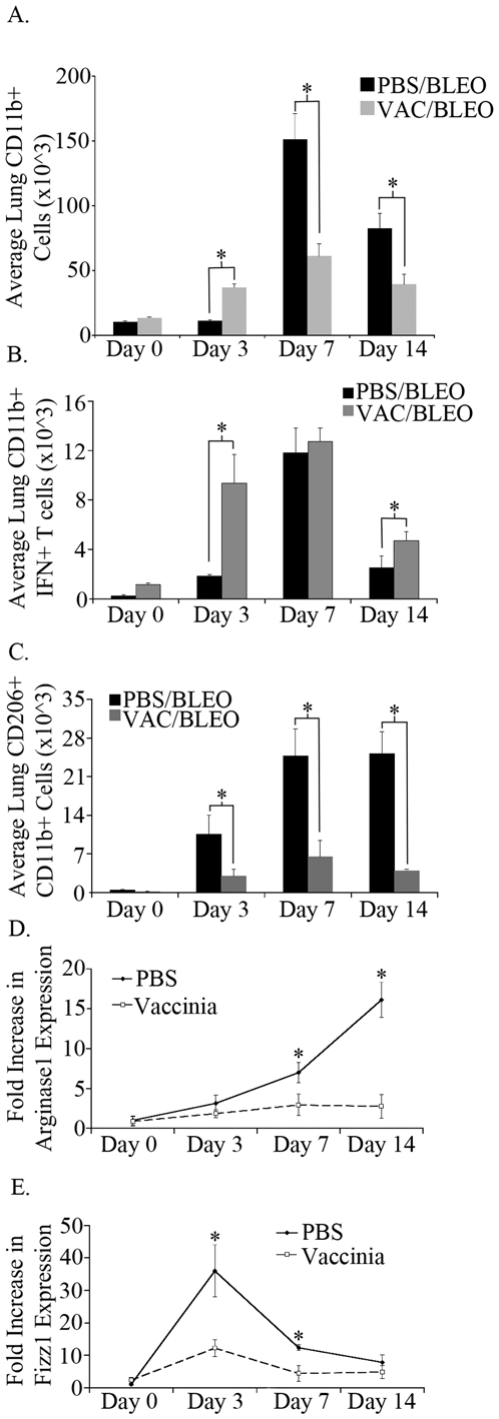
Vaccinia vaccinated mice have greater numbers of lung M1 macrophages and fewer lung M2 macrophages. A. Total lung CD11b^+^ cells following bleomycin administration. B. Total lung CD11b^+^ IFNγ^+^ cells following bleomycin administration. C. Total lung CD11b^+^ CD206^+^ cells following bleomycin administration. D. Arginase1 RNA expression from lung tissue. E. Fizz1 RNA expression from lung tissue. Error bars indicate one standard deviation of the mean and (*) indicates statistical significance (p<.05) between groups. All experiments were performed at least three times, at least 10 animals per group per experiment.

First, we analyzed the effect of vaccinia vaccination on macrophage recruitment upon bleomycin exposure. C57BL/6 mice were vaccinated as previously described and bleomycin was instilled intratracheally seven days later. On days 0, 3, 7, and 14 following bleomycin challenge lungs were isolated, processed to single cell suspensions and stained for the presence of CD11b^+^ macrophages (Figure 3A). As was seen with T cells in the lungs, the number of macrophages was not significantly changed following vaccinia treatment prior to bleomycin challenge when compared to mock (PBS) vaccinated mice at day 0. After bleomycin, the vaccinia treated mice did display an early significant increase in CD11b^+^ lung cells, however macrophage numbers did not increase significantly on days 7 and 14. Mock (PBS) vaccinated mice had significantly increased lung CD11b^+^ macrophages on day 7 and day 14 after bleomycin, approximately 2 fold more than vaccinia treated mice. Overall, while vaccinia treated mice had an earlier increase in macrophages in the lung after bleomycin, later time points did not show protracted total macrophage numbers when compared to mock (PBS) vaccinated mice that received bleomycin.

Next, we examined the effect of vaccinia vaccination on macrophage subsets post bleomycin exposure. To examine lung M1 macrophages we performed ICS for IFNγ on CD11b^+^ macrophages on days 0, 3, 7, and 14 following bleomycin challenge on both mock (PBS) vaccinated and vaccinia vaccinated mice (Figure 3B). Lungs of vaccinia treated mice had increased numbers of IFNγ^+^ CD11b^+^ macrophages prior to bleomycin challenge on day 0, indicating that vaccinia vaccination alone was activating some macrophages. There were significantly more IFNγ^+^ CD11b^+^ macrophages on day 3 in the lungs of vaccinia treated mice when compared to mock (PBS) vaccinated mice, however by day 7 the numbers of lung IFNγ^+^ CD11b^+^ macrophages were not significantly different between the two groups. Similar to CD4^+^ and CD8^+^ T cells, vaccinia treated mice maintained a higher number of IFNγ^+^ CD11b^+^ macrophages on day 14 than mock (PBS) vaccinated. Overall, vaccinia treatment induced an earlier population of IFNγ^+^ CD11b^+^ macrophages, and maintained a larger population of these cells at day 14.

Recently the mannose receptor (CD206) has been shown to be a surface marker for M2 macrophages in multiple *in vivo* models [24]. Therefore, we chose to analyze the expression of CD206 on macrophages following bleomycin challenge on both mock (PBS) vaccinated and vaccinia vaccinated mice (Figure 3C). On days 3, 7, and 14 mock (PBS) vaccinated mice displayed significantly increased numbers of CD206^+^ CD11b^+^ cells whereas vaccinia treated mice displayed relatively low levels of CD206^+^ CD11b^+^ macrophages. In addition to CD206, we analyzed the expression of other markers of M2 macrophages. To accomplish this RNA was isolated from lungs of mice on days 0, 3, 7 and 14 following bleomycin administration. Real time PCR was performed on lung cDNA to determine the expression of the M2 markers Arg1 and Fizz1 (Figures 3D, E). RNA for both Arg1 and Fizz1 was significantly increased in PBS treated mice which received bleomycin whereas Arg1 and Fizz1 expression remained low in vaccinia vaccinated mice. These data indicate that M2 macrophages are present at high levels following bleomycin challenge and that vaccinia vaccination suppresses the development of these cells. Overall, vaccinia vaccination promotes an M1 phenotype in lung macrophages following bleomycin challenge. That is, intranasal vaccinia vaccination prior to bleomycin exposure creates an immune microenvironment that fosters the generation of M1 macrophages rather than M2 macrophages upon bleomycin exposure.

### Vaccinia vaccination abrogates lung fibrocyte accumulation in bleomycin-induced pulmonary fibrosis

**Figure 4:**
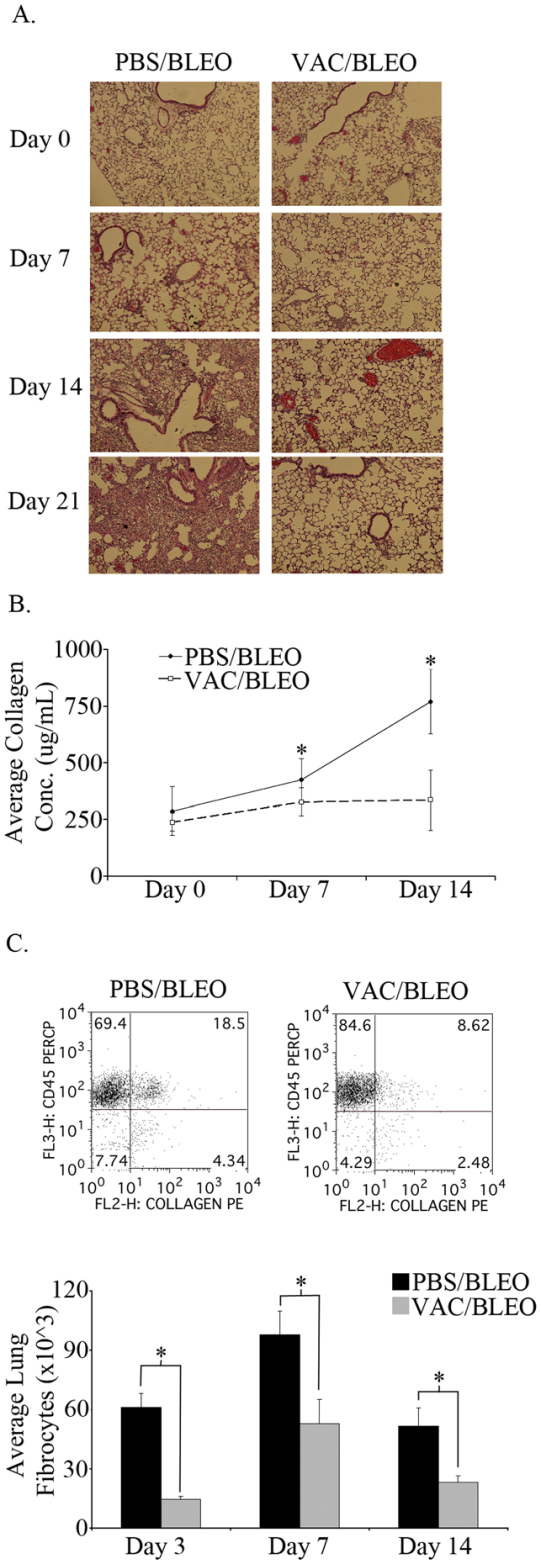
Vaccinia vaccination abrogates bleomycin induced pulmonary fibrosis. A. Histological analysis of lungs of vaccinia vaccinated and mock (PBS) vaccinated mice following bleomycin administration. B. Total lung collagen concentration following bleomycin administration. C. Flow cytometry and graphical analysis of fibrocyte (CD45^+^, Collagen^+^) numbers in lungs of mice 7 days after bleomycin administration. Error bars indicate one standard deviation of the mean and (*) indicates statistical significance (p<.05) between groups. All experiments were performed at least three times, at least 10 animals per group per experiment.

Intratracheal bleomycin induces pulmonary fibrosis through the deposition of collagen that distorts the architecture of the lung and compromises respiratory function. We observed that vaccinia vaccination enhanced survival and maintenance of weight following bleomycin challenge (figure 1). Interestingly, vaccination did not inhibit inflammation (inflammatory cell recruitment to the lungs was not inhibited) but rather vaccination promoted the induction of a robust Th1 response. We next wanted to determine the mechanism by which Th1 cells might prevent fibrosis. Recently, CD45^+^ collagen^+^ fibrocytes have been implicated in playing an important role in elaboration of extracellular matrix leading to pulmonary fibrosis [25], [26]. In vitro, it has been demonstrated that Th1 responses inhibit fibrocyte differentiation [27]. Thus, we wanted to determine if vaccinia vaccination inhibited fibrocyte accumulation in the lungs. To address this, C57BL/6 mice were vaccinated with vaccinia or mock vaccinated with PBS and seven days later bleomycin was instilled intratracheally. Lungs were isolated and histology was performed on days 0, 7, and 21 (Figure 4A). Prior to bleomycin challenge, the lungs of vaccinia vaccinated mice displayed normal architecture with no significant inflammatory cell infiltrate, indicating that the dose of vaccinia utilized did not alter lung architecture or function. Such findings were consistent with the Day 0 FACS analysis of BAL fluid (Figure 2). Seven days after bleomycin challenge the lungs of mock (PBS) vaccinated mice displayed peribronchial monocytic and lymphocytic infiltrate and some scant hemorrhage and alveolar infiltration. In comparison, the lungs of vaccinia vaccinated mice had drastically reduced peribronchial infiltrate. Fibrocytes are differentiated from bone marrow derived monocytes and histological analysis of the lungs from vaccinated mice demonstrated decreased recruitment of hematopoietically-derived cells. Furthermore, lungs of day 21 mock (PBS) vaccinated mice displayed dense cellular consolidation with protein deposition, interstitial thickening and airspace effacement, all characteristic of bleomycin-induced pulmonary fibrosis. In contrast, lungs of vaccinia vaccinated mice displayed dramatically reduced peribronchial and airspace infiltration without significant consolidation or protein deposition. In addition, we wanted to examine the deposition of collagen in the vaccinia and mock (PBS) vaccinated mice. To accomplish this, lungs were isolated 3, 7, and 14 days following bleomycin challenge with or without vaccinia vaccination, and total lungs were subjected to hydroxyproline analysis (Figure 4B). There were similar levels of collagen in vaccinia vaccinated and mock (PBS) vaccinated mice on days 3 and 7. However on day 14 the collagen levels of vaccinia vaccinated mice remained constant whereas the collagen levels of mock (PBS) vaccinated mice increased two fold. These histologic data demonstrate that the ability of vaccinia vaccination to protect against bleomycin-induced pulmonary fibrosis is associated with interruption of the late (persistent) inflammation leading to fibrosis.

In as much as fibrocytes are believed to play an important role in chronic inflammation leading to fibrosis, we next directly examined the effect of vaccinia vaccination on lung fibrocyte accumulation post-bleomycin exposure. Lung single cell suspensions were stained with CD45 and collagen and analyzed by flow cytometry (Figure 4C). Mock (PBS) vaccinated mice had a significantly higher percentage of fibrocytes in the lung following bleomycin when compared to the vaccinia treated mice on day 7. In addition, total fibrocyte numbers were significantly greater at days 3, 7, and 14 following bleomycin in the mock (PBS) vaccinated mice.

### T cell specific inhibition of fibrosis

**Figure 5:**
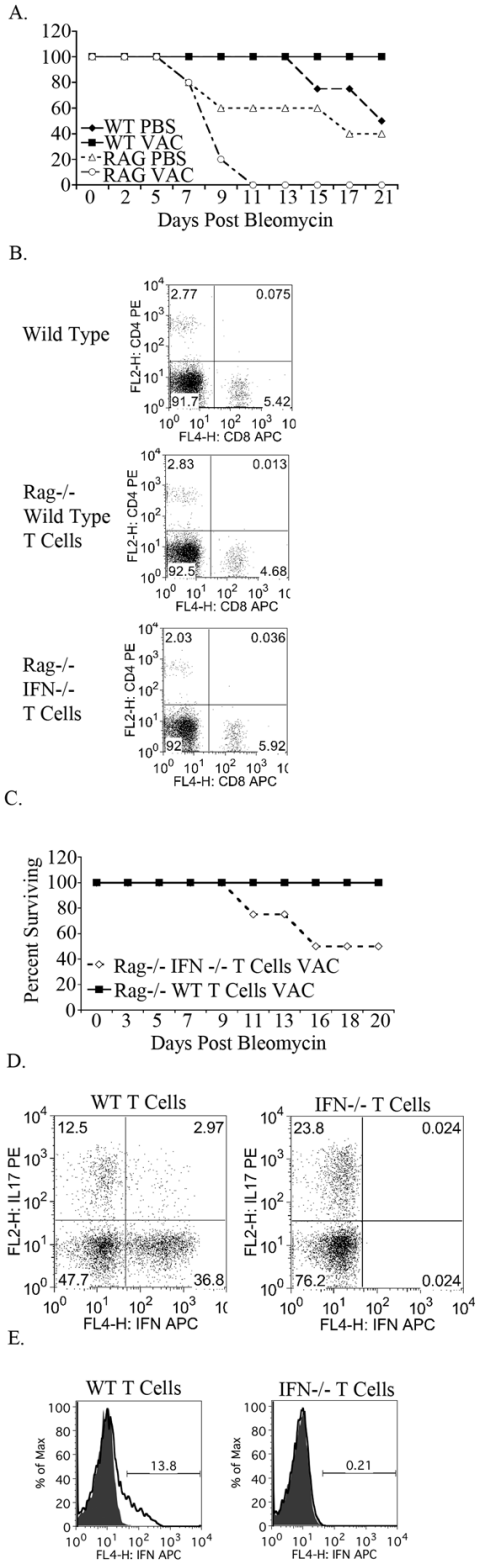
T cell derived IFNγ is necessary for protection from bleomycin induced fibrosis. A. Survival curves of wild type and Rag null mice following vaccinia vaccination or mock (PBS) vaccination and bleomycin administration. B. Flow cytometric analysis of T cell reconstitution of Rag null mice. C. Survival curves of Rag null mice reconstituted with either wild type of IFNγ null CD4^+^ and CD8^+^ T cells. All mice were vaccinated with vaccinia and bleomycin was administered. D. Flow cytometric analysis of lung CD4^+^ IFNγ^+^ and CD4^+^ IL17^+^ T cells. E. Flow cytometric analysis of lung CD11B^+^ IFNγ^+^ cells. Histograms are gated on CD11b^+^ cells. All experiments were performed at least three times, at least 10 animals per group per experiment.

Immunotherapy in the form of vaccinia vaccination leads to decreased M2 macrophages, decreased fibrocytes and ultimately decreased fibrosis upon bleomycin induced lung injury. We next wanted to definitively demonstrate that this protective effect was indeed due to the vaccinia-induced T cell response. To test this, bleomycin was administered to mice lacking T cells in combination with mock (PBS) vaccine or vaccinia vaccine. Wild type and Rag null C57BL/6 mice were either mock (PBS) vaccinated or vaccinated with vaccinia, and seven days later bleomycin was administered intranasally. Mouse survival was recorded over three weeks (Figure 5A). Wild type mice that received vaccinia prior to bleomycin were protected from fibrosis and had 100% survival while only 50% of wild type mice that were mock (PBS) vaccinated survived. Rag null mice that received a mock (PBS) vaccination prior to bleomycin displayed earlier mortality than wild type mock (PBS) vaccinated mice and decreased survival overall. Interestingly, Rag null mice which received vaccinia prior to bleomycin had 100% mortality by day 11, indicating that in the absence of T cells vaccinia induces lethal inflammation in the lung. Overall, these data support our hypothesis that the inhibition of fibrosis observed in our model is T cell driven.

Next we wanted to demonstrate that it was specifically the vaccinia-induced Th1 response that prevented fibrosis. To this end, Rag mice were reconstituted with 2 million wild type CD4^+^ T cells and 2 million wild type CD8^+^ T cells or 2 million IFNγ null CD4^+^ T cells and 2 million IFNγ null CD8^+^ T cells. Seven days after the T cell transfer flow cytometry for CD4^+^ and CD8^+^ T cells was performed on tail blood to ensure equivalent T cell reconstitution (Figure 5B). Transferred wild type and IFNγ null T cells were equally efficient in reconstituting the T cell compartment of Rag null mice. Further, T cell reconstitution mimicked CD4 and CD8 ratios seen in wild type C57B6 mice. All mice were subsequently vaccinated with vaccinia and then 7 days later received intratracheal bleomycin. Vaccinia vaccinated Rag null mice that received wild type CD4^+^ and CD8^+^ T cells had 100% survival following bleomycin challenge (Figure 5C). However, vaccinated Rag null mice that received IFNγ null CD4^+^ and CD8^+^ T cells demonstrated only 50% survival. That is in the absence of T cell derived IFN-γ, the vaccine was ineffective. This is in spite of the fact that other bone marrow derived cells are IFN-γ competent.

Next we correlated IFNγ dependent survival with macrophage and T cell function. Cells were isolated from the lungs of mice that had received wild type or IFN-γ null T cells and analyzed on day 7. CD4^+^ T cells from mice which received IFNγ null T cells had twice the percentage of IL17^+^ CD4^+^ T cells than mice which received wild type T cells (Figure 5D). That is, vaccinia-induced T cell derived IFN-γ inhibited the generation of the Th17 response in the lungs of mice treated with bleomycin. When we examined macrophages, we found that the vaccinia-induced Th1 response also played an important role in skewing toward a protective M1 macrophage response. Indeed, vaccinia vaccinated mice that were reconstituted with IFN-γ null T cells, failed to generate M1 macrophages.

### Vaccinia vaccination provides long term resistance to bleomycin induced pulmonary fibrosis

**Figure 6:**
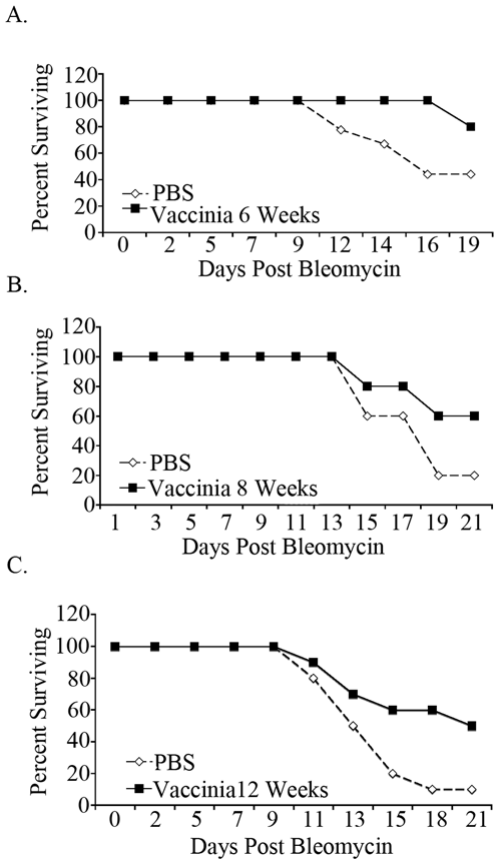
Vaccinia vaccination provides long term protection against bleomycin induced pulmonary fibrosis. Survival curves of mice vaccinia vaccinated or mock (PBS) vaccinated either six weeks (A.) eight weeks (B.) or twelve weeks (C.) prior to bleomycin administration. All experiments were performed at least three times, at least 10 animals per group per experiment.

Our data demonstrate that intranasal vaccinia vaccination 7 days prior to bleomycin exposure induces a Th1 response in the lungs that is able to mitigate the dysregulated inflammation that leads to fibrosis. Such findings provide important insight in terms of the role of Th1 T cells in regulating lung inflammation. However, we also wanted to know the potential duration of the vaccinia effect. To address this, C57BL\6 mice were given mock (PBS) or vaccinia vaccine and then exposed to bleomycin 6, 8, and 12 weeks later (Figure 6). In each instance, mice that received vaccinia vaccination had greater survival than mock (PBS) vaccinated mice. That is, intranasal vaccinia vaccination mitigated death due to pulmonary fibrosis even when there was 3 months between the vaccine and bleomycin exposure. Interestingly, as times between vaccination and bleomycin challenge increased so did the overall mortality in response to bleomycin. We believe that this finding is consistent with the observation that older mice are more susceptible to bleomycin-induced pulmonary fibrosis [28]. Nonetheless, in spite of the overall increase in mortality in the older mice, the vaccinia vaccination was still able to mitigate disease.

## Discussion

Pulmonary fibrosis is a common, often fatal, poorly treatable final end point for a diverse group of pulmonary diseases. It is characterized by the recruitment and proliferation of fibroblasts, lung myofibroblasts, epithelial-mesenchymal transition and bone marrow derived fibrocytes leading to the deposition of excess extracellular matrix and the subsequent destruction of the lung parenchyma. This process is driven by dysregulated chronic inflammation leading to the elaboration of profibrotic chemokines and cytokines. The study of human pulmonary fibrosis has been complicated by the unknown etiology, variable natural history, advanced disease at presentation and the insidious progression of fibrosis over years and even decades. In this paper we utilized the bleomycin model of lung fibrosis. Although no animal model of lung fibrosis is perfect, the bleomycin model is a well-established, well-studied, reproducible model of murine lung fibrosis that can be interrogated to reveal potential mechanisms underlying human fibrotic lung disorders [20], [29], [30], [31]. We hypothesized that the dysregulated inflammation leading to fibrosis might be regulated by the presence of disease inhibiting T cells. Indeed, immunotherapy in the form of vaccinia vaccination mitigated lung fibrosis in the bleomycin mouse model of disease. Mechanistically, this form of immunotherapy was shown to be dependent on T cell derived IFN-γ and resulted in a marked decrease in the recruitment of M2 macrophages, fibrocytes and subsequent collagen deposition. Overall these findings provide important insight in terms of the ability of Th1 effector T cells to regulate disease as well as suggest a novel treatment approach.

In light of the fact that the lungs are constantly exposed to the outside environment, the pulmonary immune response is both varied and robust. Pro-inflammatory cytokines and chemokines are produced by not only bone marrow derived resident lung macrophages and dendritic cells, but also by epithelial cells lining the upper airways [32], [33]. This facilitates both rapid innate responses as well as the upregulation of the adaptive immune response. The development of fibrosis has generally been associated with cytokines and chemokines that are primarily associated with a Th2 type response [7]. Indeed, IL-4 and IL-13 have been described in lungs from patients with Idiopathic Pulmonary Fibrosis and contribute to the development of fibrosis in a number of mouse models [34], [35]. For example, the IL-4 receptor is present on lung fibroblasts and IL-4 has been shown to promote the deposition of extracellular matrix proteins, collagen and subsequent fibrosis [7]. Further, IL-4 can promote the generation of M2 macrophages, which can facilitate fibrosis in part through the elaboration of TGF-β and PDGF [34]. M2 macrophages have been shown to be increased in the lungs of Idiopathic Pulmonary Fibrosis patients as well as in the lungs of bleomycin treated mice [23], [36]. Indeed, in our model, intranasal vaccinia vaccination abrogated the increase in M2 macrophages upon bleomycin treatment. Recent studies have also demonstrated a role for IL-10 in both fibrocyte recruitment and alternatively activated macrophage activation in the bleomycin model [22]. Finally, IL-17 has also been shown to play a role in the pathogenesis of bleomycin-induced lung fibrosis [20]. It was found that IL-1 produced by both Th17 and γδ T cells lead to increased neutrophilia and fibrosis in lungs of bleomycin treated mice [20].

Taken together these studies emphasize the potential role of T cell mediated immunity in promoting fibrosis. Studies designed to determine the precise role of T cell effector subsets in the development of fibrosis have been confusing. Simplistic models have focused on the role of IFN-γ in preventing fibrosis. Indeed, patients with progressive Idiopathic Pulmonary Fibrosis and scleroderma-pulmonary fibrosis have decreased IFN-γ in their bronchoalveolar lavage fluid [37], [38], [39], [40]. Early clinical trials with systemic IFN-γ looked promising but ultimately failed to demonstrate efficacy possible due to off target effects of systemic delivery (for example the activation of macrophages, NK cells and neutrophils) [37], [41], [42]. By employing genetically modified mice, the contribution of Th1 and Th2 cytokines has also been investigated in the past without yielding a clear role. Using the bleomycin model there are studies demonstrating increased disease in mice that over-express the Th2 transcription factor GATA-3 while other studies show decreased disease in IL-4 over-expressing mice [4], [43]. Alternatively, there are studies showing increased disease in Th1 compromised mice (T-bet null, IL-12 null) while other studies show decreased disease or no effect on disease in IFN-γ null mice [44], [45], [46], [47]. Further, some studies have shown that treatment with IFN-γ, IP-10 or IL-12 can mitigate disease [48], [49] while others show less disease in mice treated with anti-IL-12 [50]. We believe that these conflicting results reflect the fact that systemic deletion or over-expression of cytokines can affect both the initial inflammatory insult and the chronic fibrosis-inducing process. This is seen in the studies of Pham et al. who demonstrated that IL-4 both inhibited the initial inflammation to bleomycin (without the initial inflammation there is no chronic fibrosis) and enhanced the fibrogenic process [5]. Thus with rare exceptions, these cited studies do not specifically examine the role of discrete T helper cell subsets to regulate fibrosis, but rather the global effects of cytokines without distinguishing between the effects on the initial inflammation and the chronic fibrogenic process.

Since both Th2 and Th17 mediated immune responses have been implicated in promoting the chronic inflammation leading to lung fibrosis, we hypothesized that the generation of Th1 effector responses in the lung might prevent this process. Our data demonstrate that intranasal delivery of vaccinia virus was able to prevent the development of fibrosis upon bleomycin exposure. As its name implies, vaccinia virus was developed as a means of inducing protective immunity against small pox. By employing an ova expressing vaccinia construct, we were able to demonstrate that the protection against fibrosis is commensurate with the generation of an antigen specific Th1 response in the lungs. The vaccine failed to protect when Rag^−/−^ mice were reconstituted with T cells lacking IFN-γ. Importantly, these latter findings demonstrate the specific role of T cells (and T cell derived IFN-γ) in inhibiting fibrosis. That is, in spite of the fact that all of the other cells in the lungs were capable of producing IFN-γ, protection was not observed in the absence of IFN-γ producing T cells. These observations accentuate the immunoregulatory capability of T helper cells in this model and provide a partial explanation as to why a trial employing systemic IFN-γ treatment failed to impact disease in idiopathic pulmonary fibrosis patients [51].

Data are emerging implicating an important role for bone marrow-derived fibrocytes in driving lung fibrosis [52]. Fibrocytes normally make up 0.1–1% of nucleated cells in the blood [53]. In addition to CD45 they express various chemokine receptors including CXCR4 which promotes their migration to inflamed lungs in response to CXCL12 gradients [25], [26]. In both the bleomycin model and human Idiopathic Pulmonary Fibrosis, fibrocytes are believed to play a critical role in laying down collagen leading to fibrosis [26], [54]. Levels of peripheral blood fibrocytes have been shown to correlate with disease activity in patients with idiopathic pulmonary fibrosis [55], [56]. Consistent with their role in promoting lung fibrosis, the ability of vaccinia vaccination to inhibit disease was associated with a decrease in the number of lung fibrocytes in response to bleomycin. Interestingly, Shao et al. have shown that Th1 cytokines inhibit fibrocyte differentiation while Th2 cytokines promote fibrocyte generation from peripheral blood monocytes [27]. Thus mechanistically, our data suggest that the ability of Th1 induced immunotherapy to prevent pulmonary fibrosis is in part through the inhibition of fibrocyte differentiation in the lungs.

There is currently no effective treatment for pulmonary fibrosis. Based on our findings, we propose enforced skewing of T helper cell responses as a novel form of immunotherapy to mitigate the development of lung fibrosis. Interesting, we demonstrate that a single vaccination with vaccinia was able to mitigate bleomycin-induced lung injury and death up to 12 weeks after vaccination. Such observations suggest that vaccination and subsequent enforced T helper cell skewing may have the ability to both prevent and more importantly arrest the development of fibrosis even after the initial insult.

## Materials and Methods

### Mice

All animal protocols were approved by the Institutional Animal Care and Use Committee of Johns Hopkins University (Baltimore, MD). C57BL/6, B6.129S7-Rag1tm1Mom/J, and B6.129S7-Ifngtm1Ts/J were purchased from The Jackson Laboratory (Bar Harbor, ME). C57BL/6 Thy1.1^+^, OTII^+^ mice were generated by crossing C57BL/6 Thy1.1^+^ and C57B6 OTII^+^ mice purchased from The Jackson Laboratory (Bar Harbor, ME).

### Reagent

Vaccinia was generated as previously described [57]. Bleomycin was purchased from App pharmaceuticals, (Schaumburg, IL). PMA and Ionomycin were purchased from Sigma-Aldrich (Milwaukee, WI). Flow cytometry antibodies and reagents were purchased from BD (Franklin Lakes, NJ). CD206 antibody was purchased from ABD Serotec (Raleigh, NC). Collagen antibody was purchased from Abcam (Cambridge, MA).

### Pulmonary fibrosis model

2 million vaccinia particles were administered intransally on day −7. .025 U of bleomycin was administered on day 0 by tracheal cutdown. Mice were sacrificed at various intervals, lungs were exsanguinated, removed, processed to single cell suspensions, and stimulated in vitro with PMA and Ionomycin.

### Flow cytometry

All flow cytometry was performed on a FacsCaliber, BD Biosciences (San Jose, CA).

### ELISA

IFNγ ELISA was performed according to manufacturer's protocol, ebioscience (San Jose, CA).

### Collagen assay

Hydroxyproline assay was performed according to manufacturer's protocol, biovision (San Francisco, CA).

### Histology

Lungs were inflated to pressure with formalin, sectioned and stained for H&E. Samples were analyzed by microscope at 10× magnification.

### Real Time PCR

RNA was isolated using Trizol reagent, Invitrogen (Carlsbad, CA). Real Time PCR was performed using reagents purchased from Applied Biosystems. Real Time PCR was performed on a 7500 system Applied Biosystems (Carlsbad, CA).

### Statistical analysis

All statistical analysis was conducted using the paired Student's t-test. Statistical significant values were those where p<0.05.

## Supporting Information

Figure S1 **Intranasal vaccinia administration induces T cell recruitment to the lung.** Thy1.1^+^ OT-II^+^ T cells were transferred intravenously into wild type recipients. The next day mice received 2×10^∧6^ vaccinia viral particles or PBS intranasally. Three days later lungs were isolated, processed to single cell suspensions and flow cytometry was performed in order to determine percentages of Thy1.1^+^ OT-II^+^ lung T cells. All experiments were performed at least three times, at least 10 animals per group per experiment.
(TIF)
