# Supplementary figures and images for: Pulmonary Vaccination as a Novel Treatment for Lung Fibrosis
Source: PLoS One. 2012 Feb 17;7(2):e31299. doi: 10.1371/journal.pone.0031299 (PMC3281950; doi:10.1371/journal.pone.0031299)

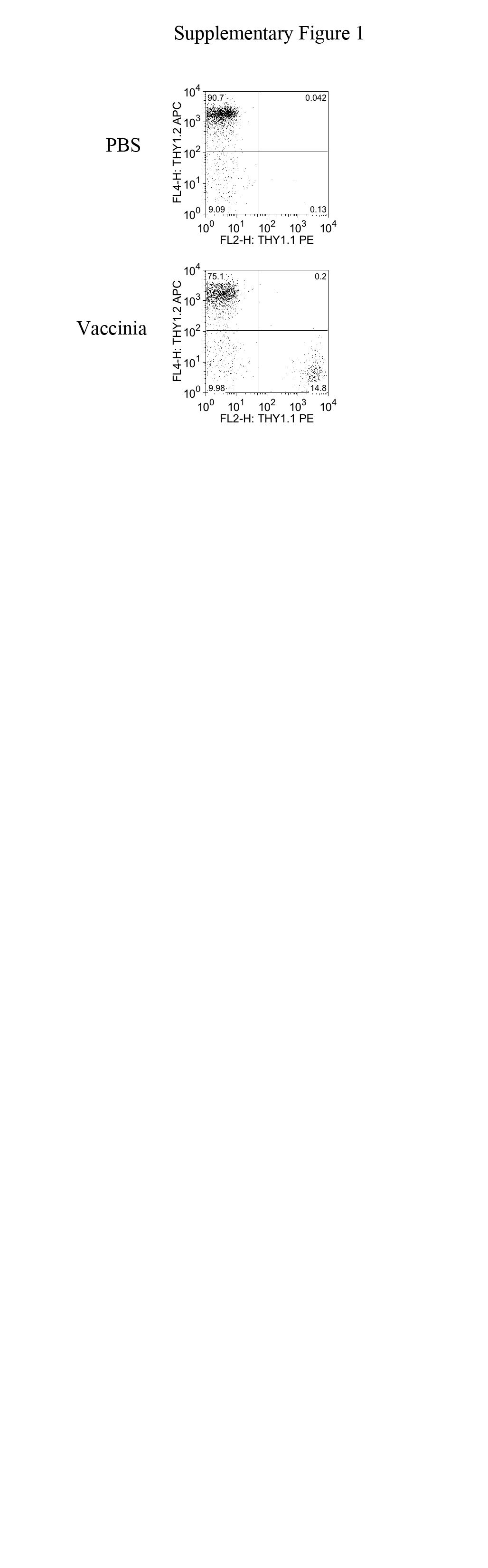

Supplement: Figure S1 — Intranasal vaccinia administration induces T cell recruitment to the lung. Thy1.1+ OT-II+ T cells were transferred intravenously into wild type recipients. The next day mice received 2×10∧6 vaccinia viral particles or PBS intranasally. Three days later lungs were isolated, processed to single cell suspensions and flow cytometry was performed in order to determine percentages of Thy1.1+ OT-II+ lung T cells. All experiments were performed at least three times, at least 10 animals per group per experiment. (TIF) [file pone.0031299.s001.tif]
